# Ubc1 turnover contributes to the spindle assembly checkpoint in *Saccharomyces cerevisiae*

**DOI:** 10.1093/g3journal/jkab346

**Published:** 2021-09-29

**Authors:** Heather E Arsenault, Julie M Ghizzoni, Cassandra M Leech, Anne R Diers, Stephane Gesta, Vivek K Vishnudas, Niven R Narain, Rangaprasad Sarangarajan, Jennifer A Benanti

**Affiliations:** 1 Department of Molecular, Cell and Cancer Biology, University of Massachusetts Medical School, Worcester, MA 01605, USA; 2 BERG, Framingham, MA 01710, USA

**Keywords:** mitosis, anaphase-promoting complex, spindle assembly checkpoint

## Abstract

The spindle assembly checkpoint protects the integrity of the genome by ensuring that chromosomes are properly attached to the mitotic spindle before they are segregated during anaphase. Activation of the spindle checkpoint results in inhibition of the Anaphase-Promoting Complex (APC), an E3 ubiquitin ligase that triggers the metaphase–anaphase transition. Here, we show that levels of Ubc1, an E2 enzyme that functions in complex with the APC, modulate the response to spindle checkpoint activation in *Saccharomyces cerevisiae*. Overexpression of Ubc1 increased resistance to microtubule poisons, whereas Ubc1 shut-off sensitized cells. We also found that Ubc1 levels are regulated by the spindle checkpoint. Checkpoint activation or direct APC inhibition led to a decrease in Ubc1 levels, charging, and half-life. Additionally, stabilization of Ubc1 prevented its down-regulation by the spindle checkpoint and increased resistance to checkpoint-activating drugs. These results suggest that down-regulation of Ubc1 in response to spindle checkpoint signaling is necessary for a robust cell cycle arrest.

## Introduction

Accurate progression through the cell cycle depends upon the properly timed synthesis and degradation of hundreds of regulatory proteins. One key regulator of protein degradation during the cell cycle is the Anaphase-Promoting Complex (APC), which is an essential E3 ubiquitin ligase that ubiquitinates proteins to target them for degradation in the proteasome (Morgan 2007). The APC targets its substrates during mitosis and G1 phase, coordinating orderly progression through these stages of the cell cycle. Degradation of some APC targets is required for cell cycle progression. The APC must ubiquitinate the mitotic inhibitor securin for cells to progress from metaphase into anaphase (Cohen-Fix *et al.* 1996; Shirayama *et al.* 1999), and B-type cyclins to allow cells to exit from mitosis and begin the next cell cycle (Irniger *et al.* 1995; King *et al.* 1995; Sudakin *et al.* 1995; Tugendreich *et al.* 1995). Because of these essential functions, regulation of the APC is of critical importance to ensure the accuracy of cell division.

The APC works with multiple E2 enzymes to add ubiquitin chains to substrates. In budding yeast, chain formation on APC substrates is initiated by Ubc4, which is followed by chain extension by a second E2, Ubc1 (Rodrigo-Brenni and Morgan 2007). Similarly, sequentially acting E2s work with the APC in metazoan cells. In human and drosophila, UbcH10 adds short chains to APC substrates, which are then extended by Ube2S (Garnett *et al.* 2009; Williamson *et al.* 2009; Wu *et al.* 2010). The human homolog of Ubc1, Ube2K can similarly extend chains *in vitro* (Rodrigo-Brenni and Morgan 2007), but is less efficient than Ube2S and its role in working with the APC *in vivo* remains unclear (Wu *et al.* 2010). Importantly, one mechanism by which APC activity is regulated during the cell cycle is by controlling the levels of these E2 enzymes that are required for APC-dependent ubiquitination. Both UbcH10 and Ube2S are themselves autoubiquitinated and degraded by the APC at the end of G1 phase, helping to ensure that APC activity is extinguished as cells enter S phase (Rape and Kirschner 2004; Williamson *et al.* 2009).

Another mechanism that controls APC activity is the spindle assembly checkpoint (SAC), which is activated when chromosomes are not properly attached to the mitotic spindle. Activation of the SAC leads to the formation of the mitotic checkpoint complex (MCC) that binds to the APC activator Cdc20, preventing degradation of securin and blocking cells at the metaphase–anaphase transition (Musacchio 2015). When all chromosomes are stably attached to the spindle, SAC-signaling is shut off, the APC is reactivated, and the cell cycle resumes. Notably, APC activity is itself required to enable cells to resume cycling following a SAC arrest (Reddy *et al.* 2007). This is due is in part to autoubiquitnation of Cdc20, which triggers disassembly of the MCC (Garnett *et al.* 2009; Foster and Morgan 2012; Uzunova *et al.* 2012).

Proper regulation of E2 enzymes is also important for cell cycle regulation by the SAC. The N-terminus of UbcH10 limits ubiquitination by the APC and is necessary for checkpoint control, since expression of a mutant lacking this domain enables cells to bypass SAC arrest (Summers *et al.* 2008). Similarly, UbcH10 overexpression overrides the SAC (Reddy *et al.* 2007). Misregulation of Ube2S also impacts checkpoint signaling. Either depletion or destabilization of Ube2S prolongs SAC arrest, indicating that its function is required for silencing the checkpoint (Garnett *et al.* 2009; Liess *et al.* 2020). These findings are of particular interest because both UbcH10 and Ube2S are up-regulated in cancer cells (Pallante *et al.* 2005; Berlingieri *et al.* 2007; Ieta *et al.* 2007; Fujita *et al.* 2009; Chen *et al.* 2010; Shen *et al.* 2013; Ayesha *et al.* 2016; Yoshimura *et al.* 2017; Li *et al.* 2018; Pan *et al.* 2018; Lin *et al.* 2019) and their overexpression may disrupt checkpoint control and contribute to cellular transformation.

Here, we investigated whether regulation and function of the yeast E2 Ubc1 are important for the SAC-mediated arrest. As with human E2s that function with the APC, we find that increasing or decreasing Ubc1 levels affects the sensitivity of cells to SAC activation. Although Ubc1 levels do not change during an unperturbed cell cycle, the level of Ubc1 decreases and its half-life is shortened upon SAC activation, suggesting that Ubc1 degradation is accelerated when the APC is inactivated during a SAC arrest. Moreover, cells expressing a stabilized allele of Ubc1 are more resistant to microtubule poisons that activate the SAC. In sum, our findings suggest that Ubc1 degradation upon spindle checkpoint activation is important for a robust cell cycle arrest.

## Materials and methods

### Yeast strains and plasmids

Yeast strains were constructed and grown using standard techniques (Rothstein 1991; Sherman 2002). All strains used were in the BY4741 genetic background. A complete list of strains can be found in Supplementary Table S1. *CDC27* was AID tagged as described (Morawska and Ulrich 2013) using the AID* Kit (a gift from Helle Ulrich; Addgene kit #1000000120). The Myc-Ubc1 strain (YHA377) was constructed by first integrating a *HIS3MX-UBC1p-13MYC* cassette upstream of the *UBC1* start codon, where the *UBC1p* sequence includes 500 base pairs upstream of the start codon. *UBC1* was then C-terminally tagged with *TAP-URA3* to generate the *HIS3MX-UBC1p-13MYC-UBC1-TAP-URA3* strain. A matched strain lacking the 13MYC sequence (YHA379) was constructed similarly.

To construct the pRS316-UBC1-V5, a *UBC1p-UBC1-3V5* synthetic gene fragment was cloned into pRS316. To construct the *ubc1-KR* allele, a synthetic gene in which all 16 lysines in UBC1 were mutated to arginine (Invitrogen) was recombined into the pRS316-UBC1-V5 plasmid using the NEBuilder HiFi DNA Assembly kit, generating pRS316-UBC1-KR-V5. To integrate *ubc1-KR* into the genome the sequence was first PCR amplified from pRS316-ubc1-KR-V5 and ligated to a *TAP-HIS3MX* PCR product using the NEBuilder HiFi DNA Assembly kit. The *ubc1-16KR-TAP-HIS3MX* sequence was then amplified by PCR and recombined into the genome using standard approaches. To construct pRS316-Myc-UBC1-TAP, *UBC1p-13MYC-UBC1-TAP* was amplified from YHA377 genomic DNA and cloned into pRS316 by HiFi Assembly. A synthetic cDNA was similarly cloned into pRS316 to generate pRS316-ubc1-C88S-TAP. All mutations were confirmed by sequencing. To construct pRS316-Ube2K-V5 and pRS316-Ube2S-V5, synthetic genes encoding yeast codon-optimized Ube2K and Ube2S were subcloned into the pRS316-UBC1-V5 plasmid, replacing *UBC1*. For Uba1 expression in yeast, the human UBA1 cDNA (Dharmacon) was subcloned pRS415-GPD1p to generate pRS415-GPD-UBA1-FLAG.

### Growth conditions and cell cycle synchronization

Cells were grown in rich medium (YM-1) with 2% dextrose or galactose at 30°C (Benanti *et al.* 2007). G1 arrests were performed by adding 10 µg/ml α-factor for 2–3 h. Where indicated, cells were then released into fresh media and α-factor was added back 45 min after release to prevent entry into a subsequent cell cycle. Mitotic cells were captured by first arresting cells in 10 µg/ml α-factor 2 h, then releasing cells into fresh medium for 60 min with the re-addition of α-factor as above. Nocodazole was added for 2 h to arrest cells in mitosis at the SAC. To induce degradation of Cdc27 in the *CDC27-AID* strain growing in liquid culture, IAA was added to a concentration of 0.5 mM for the length of time indicated in the figure legends.

### Serial dilution assays

For *GAL1* promoter (*GALp*)*-UBC1* shut-off experiments, cultures were grown in YM-1 with 2% raffinose. Cells were serially diluted 1:5 and spotted onto rich media containing 2% dextrose or galactose to modulate expression of *GALp-UBC1*. Benomyl was used at 10–20 µg/ml, as indicated. For degradation of Cdc27-AID, 1 mM IAA was added to rich plates with dextrose (YPD). Plates were grown at 30°C for 1–3 days. In plasmid rescue experiments, cells were spotted onto synthetic media lacking uracil and containing 2% dextrose or galactose, as indicated.

### Flow cytometry

A total of 0.15 OD of exponentially growing cells were fixed in 70% ethanol overnight at 4°C and stained with Sytox Green as previously described (Landry *et al.* 2012). DNA content was analyzed on a Guava easyCyte HT (Millipore) cytometer. Data analysis was performed using FlowJo software.

### Western blotting

A total of 1 OD cell pellets were processed using TCA lysis and Western blots performed as previously described (Leech *et al.* 2020). For non-reducing lysis, lysates were prepared the same way except that 4X SDS-PAGE sample buffer without β-mercaptoethanol (40% glycerol, 0.25 M Tris pH 6.8, 8% SDS, bromophenol blue) was used. Western blotting was performed with antibodies against Clb2 (sc-9071, Santa Cruz), FLAG (clone M2, F1804, Sigma), G6PDH (A9521, Sigma), MYC (clone 9E10, M5546, Sigma), PSTAIRE (P7962, Sigma), TAP (CAB1001, ThermoFisher), and V5 (R960-25, ThermoFisher). The PSTAIRE antibody recognizes a conserved sequence in CDK kinases and is used as a loading control.

To quantify Western blot data, images were collected on a ChemiDoc Imaging System (BioRad) and quantified using Image Lab software (Biorad). For all experiments, a minimum of three biological replicates was performed, with the exact number noted in the figure legends. Data were analyzed using GraphPad Prism software.

### Cycloheximide-chase assays

Cycloheximide was added to asynchronous or synchronized cells, as indicated, to a final concentration of 50 μg/ml. Samples were collected for Western blotting at the indicated number of minutes following the addition of cycloheximide.

### Doubling time measurements

Exponentially growing cells were diluted to an OD of 0.2 in YM-1 containing 2% dextrose with or without 5 µg/ml nocodazole, as indicated. Triplicate cultures were added to a 96-well plate, sealed with parafilm, and incubated in an Infinite M Nano (Tecan) plate reader at 30°C with shaking. OD_600_ readings were taken every 20 min. Doubling time was calculated using GraphPad Prism software.

## Results

**Figure 1:**
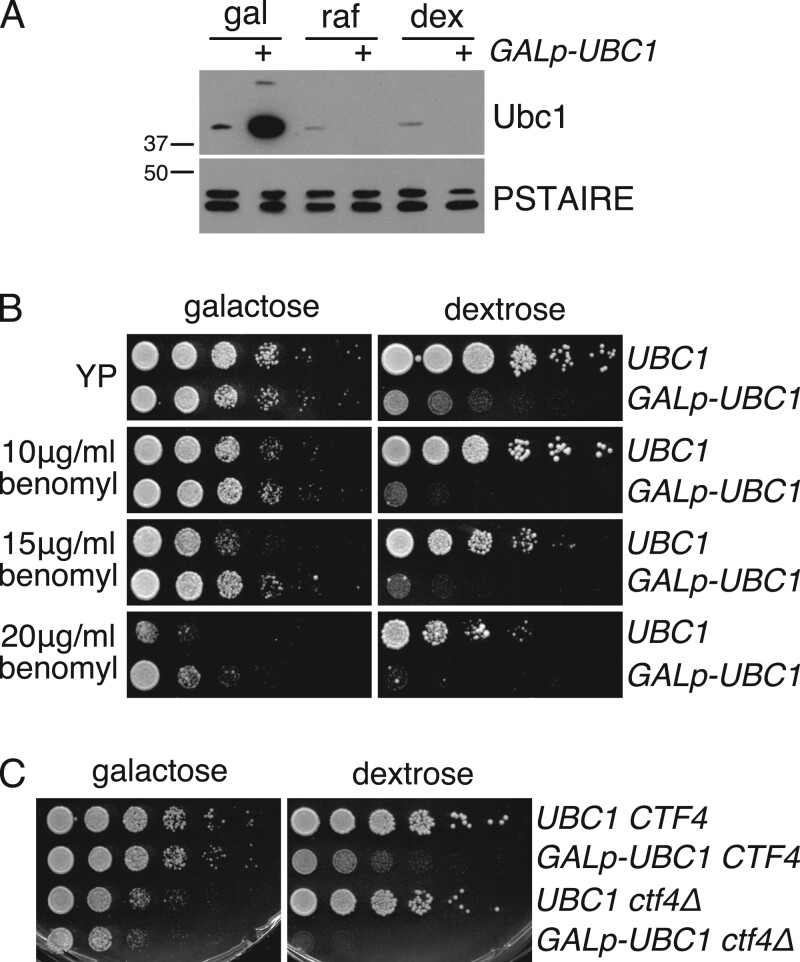
Ubc1 levels alter the response to SAC activation. (A) Expression of Ubc1 from the *GALp*. Cells expressing Ubc1-3V5 from its endogenous promoter or an integrated *GALp* were grown in galactose (gal), raffinose (raf) or dextrose (dex). Western blotting was performed with antibodies against the V5 tag on Ubc1 or PSTAIRE (loading control). (B) Fivefold dilutions of wild type (*UBC1*) or *GALp-UBC1* cells were spotted onto rich medium (YP) containing galactose or dextrose and the indicated amount of benomyl. (C) Fivefold dilutions of the indicated strains were spotted onto rich medium containing galactose or dextrose.

We investigated whether alteration of Ubc1 levels could affect the SAC response in budding yeast. To do this, we integrated the galactose-inducible *GALp* upstream of *UBC1* in the genome. In this strain, Ubc1 was overexpressed in cells growing in galactose and undetectable when cells were grown in raffinose or dextrose (Figure 1A). As previously reported (Rodrigo-Brenni and Morgan 2007), we found that *GALp-UBC1* strains grew considerably slower when Ubc1 expression was shut-off in medium containing dextrose but grew at a similar rate as wild-type cells when Ubc1 was overexpressed in medium containing galactose (Figure 1B). To examine whether changing Ubc1 levels might affect the SAC response, we re-examined the impact of Ubc1 overexpression and shut-off in the presence of sub-lethal doses of the microtubule poison benomyl. Interestingly, Ubc1 overexpression rendered cells more resistant to benomyl. Conversely, cells growing on dextrose and lacking Ubc1 were more sensitive to benomyl. Thus, modulating Ubc1 levels affects the proliferation of cells when the SAC is activated.

To examine the effect of altering Ubc1 levels in an alternate context of SAC activation, we also examined the consequence of shutting off Ubc1 expression in a *ctf4*Δ mutant. Deletion of *CTF4* was previously shown to trigger the SAC and cause a mitotic delay (Hanna *et al.* 2001), and a negative genetic interaction between *ctf4* and a temperature sensitive allele of *ubc1* was identified in a high-throughput genetic interaction screen (Costanzo *et al.* 2016). Notably, we found that Ubc1 shut-off was lethal in a *ctf4*Δ strain (Figure 1C), suggesting that Ubc1 is essential for cells to survive SAC activation.

In human cells, the E2s UbcH10 and Ube2S are both cell cycle regulated and decrease in expression when the APC is inactivated as cells enter S phase (Rape and Kirschner 2004; Williamson *et al.* 2009). Interestingly, the yeast *UBC1* mRNA has been shown to cycle with expression peaking in mitosis (Pramila *et al.* 2006), raising the possibility that Ubc1 protein levels may also cycle and contribute to cell cycle regulation. To test this, Ubc1 expression was examined over the course of the cell cycle. In addition, the charged form of Ubc1 was measured by lysing cells in non-reducing conditions to preserve the thioester bond with ubiquitin in the active site (Jin *et al.* 2007). Notably, neither total Ubc1 protein nor the percentage of charged Ubc1 changed significantly over the cell cycle (Supplementary Figure S1), suggesting that any mRNA expression changes during the cell cycle are not reflected in changes in Ubc1 protein.

**Figure 2:**
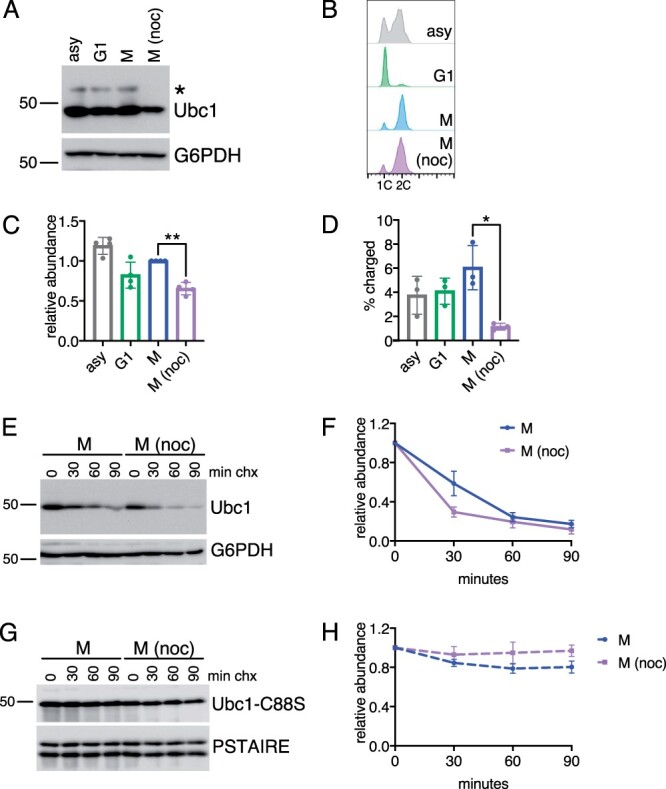
Ubc1 is down-regulated by the SAC. (A) Representative Western blot showing Ubc1-TAP levels in cells grown asynchronously (asy), arrested in G1 with α-factor (G1), synchronized in mitosis (M), or arrested in M with nocodazole [M (noc)]. All cells were lysed in non-reducing conditions to preserve the charged form of Ubc1 (*). Ubc1 was detected with anti-TAP-tag antibodies, G6PDH is shown as a loading control. (B) Flow cytometry showing DNA content in cells from (A) to confirm cell cycle position of each culture. (C) The relative abundance of total Ubc1 protein was calculated by normalizing TAP-tag signal to G6PDH. Shown are average values relative to the level in M from *n* = 4 experiments. Error bars represent standard deviations. Significance of the difference between M and M (noc) samples was calculated using a paired *t*-test, **P < 0.005. (D) The percentage of charged Ubc1 was calculated by dividing the amount of charged Ubc1 by the amount of total Ubc1 protein. Shown are average values from *n* = 3 experiments. Error bars represent standard deviations. Significance of the difference between M and M (noc) was calculated using a paired *t*-test, *P < 0.05. (E) Representative cycloheximide-chase assay comparing the degradation of Ubc1-TAP in cells synchronized in M to those arrested in nocodazole [M (noc)]. Ubc1 is detected with anti-TAP antibodies and G6PDH is shown as a loading control. (F) Quantitation of Ubc1 degradation from *n* = 4 replicates of the cycloheximide-chase assay shown in (E). Shown are average values at each time point, error bars represent standard deviations. (G) Representative cycloheximide-chase assay comparing the degradation of Ubc1-C88S-TAP in cells synchronized in M to those arrested in nocodazole [M (noc)]. Ubc1-C88S is detected with anti-TAP antibodies and G6PDH is shown as a loading control. Flow cytometry data confirming cell cycle positions are shown in Supplementary Figure S2B. (H) Quantitation of Ubc1-C88S degradation from *n* = 6 replicates of the cycloheximide-chase assay shown in (G). Shown are average values at each time point, error bars represent standard deviations.

Although these data revealed that Ubc1 levels were not cell cycle regulated under normal growth conditions, we reasoned that Ubc1 expression could potentially be altered when the APC is inactivated by the SAC. To test this possibility, Ubc1 expression was examined in cells arrested at the SAC with nocodazole. Interestingly, nocodazole-arrested cells had an ∼35% reduction in Ubc1 levels and a sixfold reduction in the percentage of charged Ubc1 when compared to cells synchronized in mitosis without the drug (Figure 2, A–D). Human UbcH10 and Ube2S undergo increased autoubiquitination and degradation when the APC cannot target substrates (Williamson *et al.* 2009; Liess *et al.* 2019, 2020), raising the possibility that Ubc1 may undergo similar regulation when the APC is inactivated by the SAC. Consistent with this model, the observed decrease in Ubc1 protein correlated with decreased Ubc1 half-life in nocodazole-arrested cells (Figure 2, E and F). To test whether Ubc1 may be degraded by autoubiquitination, we constructed a *UBC1* allele with the catalytic cysteine mutated to serine (Ubc1-C88S). Since Ubc1-C88S is not functional and does not support growth when expressed in the absence of the wild-type protein (Supplementary Figure S2), we expressed Ubc1-C88S from a plasmid in otherwise wild-type cells and examined its degradation. Notably, Ubc1-C88S was almost completely stable both in mitosis and upon SAC activation, supporting the model that Ubc1 is degraded via autoubiquitination (Figure 2, G and H). Thus, spindle checkpoint activation leads to reduced Ubc1 protein, a decrease in charged Ubc1 and accelerated Ubc1 degradation.

**Figure 3:**
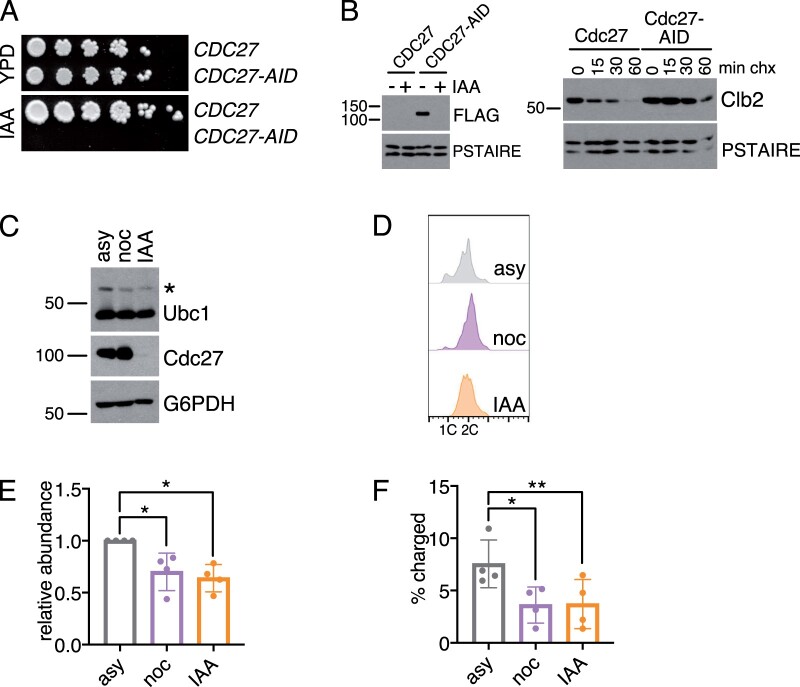
APC inactivation leads to down-regulation of Ubc1. (A) Fivefold dilutions of wild-type cells (*CDC27*) and cells expressing *CDC27-FLAG-AID* were plated on rich medium (YPD) or rich medium containing IAA. (B) Cells from (A) were treated with or without (+/−) IAA for 30 min and levels of Cdc27-FLAG-AID were detected by Western blotting against the FLAG tag. PSTAIRE is shown as a loading control (left panel). After 30 min of IAA treatment, a cycloheximide-chase assay was performed and levels of Clb2 and PSTAIRE (loading control) were detected by Western blot (right panel). (C) Representative Western blot showing levels of Ubc1-TAP, Cdc27-FLAG-AID, and G6PDH in *CDC27-AID* cells growing asynchronously (asy) or treated with nocodazole (noc) or IAA for 3 h. Blots were performed with antibodies against TAP (Ubc1), FLAG (Cdc27), and G6PDH (loading control). The asterisk indicates the charged form of Ubc1. (D) Flow cytometry plots showing DNA content of cells in (C). (E) The relative abundance of Ubc1 in cells from (C) was calculated by normalizing TAP-tag signal to G6PDH. Shown are average values relative to the level in asynchronous cells (asy) from *n* = 4 experiments. Error bars represent standard deviations. Significance of the difference between the indicated samples was calculated using a paired *t*-test, *P < 0.05. (F) The percentage of charged Ubc1 in cells from (C) was calculated by dividing the amount of charged Ubc1 by the amount of total Ubc1 protein. Shown are average values from *n* = 4 experiments. Error bars represent standard deviations. Significance of the difference between the indicated samples was calculated using a paired *t*-test, *P < 0.05, **P < 0.005.

Nocodazole is a microtubule poison that prevents microtubule polymerization and triggers the SAC by disassembling the mitotic spindle so that the mitotic chromosomes remain unattached. The ultimate consequence of the SAC is inactivation of the APC to arrest cells at the metaphase–anaphase transition (Morgan 2007). However, it is possible that changes in Ubc1 expression that occur in nocodazole-treated cells are a consequence of microtubule depolymerization and not APC inactivation specifically. To test this directly, we constructed a yeast strain that can be used to rapidly inactivate the APC by fuzing an Auxin-Inducible Degron (AID) tag to the essential APC subunit Cdc27 (Morawska and Ulrich 2013). The growth of this strain was completely inhibited in the presence of IAA (Figure 3A). Moreover, Cdc27 was completely degraded and the APC target Clb2 stabilized within 30 min of IAA addition (Figure 3B), confirming that Cdc27 degradation inhibits the APC.

We next used the Cdc27-AID strain to determine how APC inactivation affected Ubc1 levels and charging. Cdc27-AID cells were treated with nocodazole or IAA to trigger the SAC or degrade Cdc27, respectively. Levels and charging of Ubc1 were then quantified. Importantly, inactivation of the APC via Cdc27 degradation led to a similar decrease in Ubc1 protein levels and charging compared to nocodazole treatment (Figure 3, C, E, and F). IAA-treated cells were also arrested with a 2C DNA content, similar to nocodazole-treated cells, as expected (Figure 3D). Although Ubc1 levels decreased by ∼25% in Cdc27-AID cells following nocodazole addition or IAA treatment, the half-life of Ubc1 was not significantly reduced in IAA-treated cells (Supplementary Figure S3). The subtler effect on Ubc1 levels and half-life in the Cdc27-AID strain compared to a wild-type strain may be a consequence of the fact that the Cdc27-AID expressing cells are likely to have partially compromised APC function prior to IAA treatment. This is evident by the fact that the untreated population of Cdc27-AID cells has a decreased fraction of cells in G1 and an increased fraction of cells in mitosis compared to wild-type cells (compare Figures 2B to Figure 3D). However, these data are consistent with the model that APC inactivation promotes decreased Ubc1 charging and levels.

**Figure 4:**
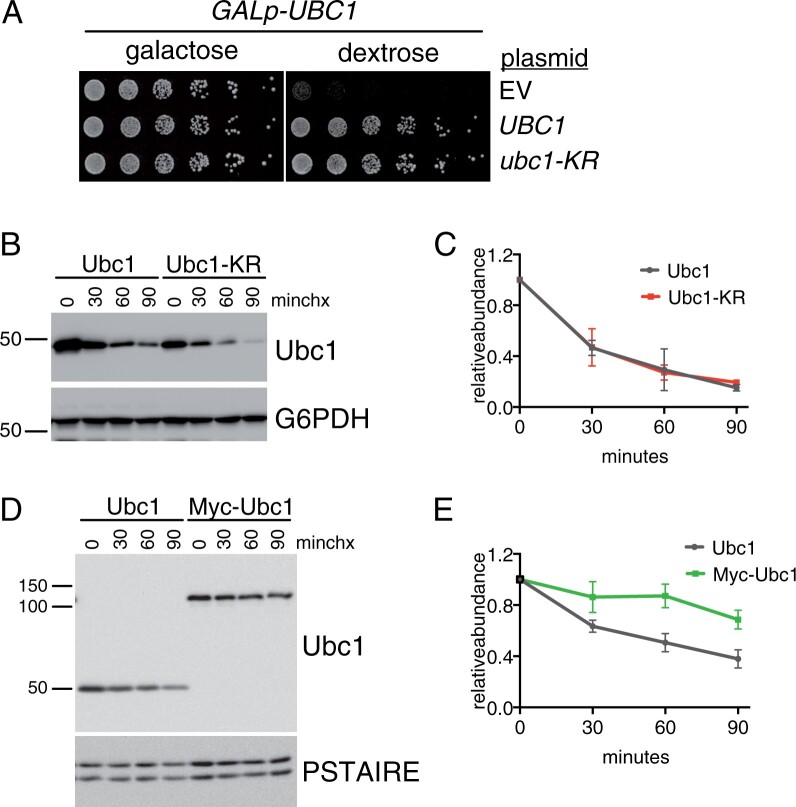
Construction of a stable allele of Ubc1. (A) Fivefold dilutions of *GALp-UBC1* cells expressing an empty vector, or plasmids expressing wild-type *UBC1* or *ubc1-KR* from the endogenous *UBC1* promoter were plated on media containing galactose or dextrose. (B) Representative cycloheximide-chase assay of cells expressing Ubc1-TAP or Ubc1-KR-TAP from the endogenous *UBC1* locus. Ubc1 is detected by the TAP tag, G6PDH is shown as a loading control. (C) Quantitation of cycloheximide-chase assays, as in (B). An average of *n* = 3 experiments is shown. Error bars represent standard deviations. (D) Representative cycloheximide-chase assay of Ubc1-TAP and 13Myc-Ubc1-TAP. Ubc1 is detected by a C-terminal TAP tag on each protein, PSTAIRE is shown as a loading control. (E) Quantitation of cycloheximide-chase assays as in (D). An average of *n* = 6 experiments is shown. Error bars represent standard deviations.

Our data show that Ubc1 is destabilized and down-regulated during the SAC and that overexpression of Ubc1 renders cells more resistant to SAC activation. Together, these findings suggest that the accelerated degradation of Ubc1 may contribute to the SAC-induced arrest. To test this hypothesis directly, we set out to create an allele of Ubc1 that retains its catalytic activity but cannot be degraded. Ubc1 has been shown to autoubiquitinate and assemble a ubiquitin chain on lysine 93 (Hodgins *et al.* 1996) and a high-throughput study identified six lysine residues in Ubc1 that are ubiquitinated (Swaney *et al.* 2013). These observations suggest that the addition of a ubiquitin chain to a lysine residue might trigger Ubc1 degradation. For this reason, we first attempted to block degradation by changing all 16 lysine residues in Ubc1 to arginines (Ubc1-KR). If lysine ubiquitination is required for Ubc1 turnover, this mutant should be stable. Surprisingly, mutation of lysine residues did not impair Ubc1 function, since the mutant protein was able to restore growth to an Ubc1 shut-off strain (Figure 4A). In addition, although it was expressed at a slightly reduced level, Ubc1-KR was degraded with similar kinetics as the wild-type protein (Figure 4, B and C). This result demonstrates that lysine residues are not required for Ubc1 turnover. Next, we considered the possibility that the N-terminus of Ubc1 could be required for its degradation. The addition of an N-terminal Myc tag stabilizes UbcH10 (Rape and Kirschner 2004), so we took a similar approach and added an N-terminal 13Myc tag to Ubc1. When compared to wild-type Ubc1, the N-terminal tag almost completely blocked Ubc1 degradation (Figure 4, D and E), suggesting that the N-terminus of Ubc1 is required for its degradation.

**Figure 5:**
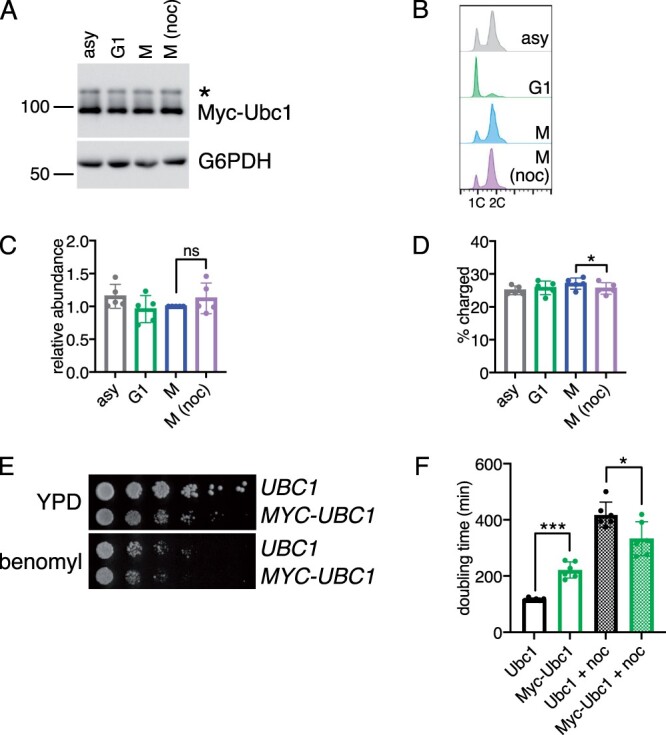
Stabilization of Ubc1 increases resistance to SAC-activating drugs. (A) Representative Western blot showing levels of 13Myc-Ubc1-TAP in cells growing asynchronously (asy), arrested in G1 with α-factor (G1), synchronized in M, or arrested in M (noc). G6PDH is shown as a loading control. (B) Flow cytometry plots of DNA content showing the cell cycle position of cells from (A). (C) The relative abundance of total Ubc1 protein was calculated by normalizing TAP-tag signal to G6PDH. Shown are average values relative to the level in M from *n* = 5 experiments. Error bars represent standard deviations. A paired *t*-test was used to compare M and M (noc) samples and the difference was found to be not significant (ns). (D) The percentage of charged Ubc1 was calculated by dividing the amount of charged Ubc1 by the amount of total Ubc1 protein. Shown are average values from *n* = 5 experiments. Error bars represent standard deviations. Significance of the difference between M and M (noc) was calculated using a paired *t*-test, *P < 0.05. (E) Fivefold dilutions of cells with the indicated genotypes were plated on rich medium (YPD) or rich medium containing 15 μg/ml benomyl. (F) Average doubling time of *UBC1 and MYC-UBC1* strains with or without 5 μg/ml nocodazole (noc) from *n* = 6 experiments. Error bars represent standard deviations. Significance of the differences between the indicated samples were calculated using a paired *t*-test, ***P < 0.0005, *P < 0.05.

We next set out to determine how stabilization of Ubc1 affects its regulation and function. First, Myc-Ubc1 levels and charging were examined in nocodazole-arrested cells and compared to cells synchronized in mitosis without the drug. Notably, Myc-Ubc1 levels did not decrease when cells were arrested with nocodazole (Figure 5A–C), confirming that stabilization of Ubc1 prevented SAC-mediated degradation. In addition, charging was largely unchanged in nocodazole-treated cells compared to mitotic cells (Figure 5, A and D). Interestingly, the addition of the N-terminal tag increased the overall fraction of charged Ubc1, from 4%–6% in wild-type cells (Figure 2D) to 24–27% in Myc-Ubc1 cells (Figure 5D), demonstrating that an increase in charged Ubc1 correlates with decreased turnover.

Finally, we examined how stabilization of Ubc1 impacted growth arrest in response to SAC activation. Myc-Ubc1 cells grew slower than control cells under standard growth conditions (Figure 5, E and F) and displayed an increased fraction of cells with a 2C DNA content (compare Figure 5B to Figure 2B). However, despite the slower proliferation rate of Myc-Ubc1 cells compared to matched control cells, Myc-Ubc1 cells were more resistant to microtubule poisons. When cells were plated on solid medium without drug Myc-Ubc1 cells grew slower than controls, however, they grew at similar rates in the presence of benomyl (Figure 5E). Moreover, when doubling times were measured in liquid culture, Myc-Ubc1 cells doubled faster in the presence of nocodazole compared to controls (Figure 5F). Thus, similar to what was observed upon Ubc1 overexpression, stabilization of Ubc1 provided resistance to SAC arrest following microtubule disruption. These data suggest that degradation of Ubc1 during the SAC is important to elicit a robust cell cycle arrest.

## Discussion

Here, we examined how the E2 enzyme Ubc1 contributes to SAC-mediated cell cycle arrest in budding yeast. Our findings show that Ubc1 is destabilized and down-regulated in response to SAC-signaling (Figure 2) and that this down-regulation contributes to growth arrest, since either overexpression or stabilization of Ubc1 provides resistance to SAC-activating drugs (Figures 1 and 5). Although E2s that partner with the APC have been shown to play a part in the SAC in metazoan cells, to our knowledge increased degradation of an E2 enzyme during the SAC has not been demonstrated before and represents an additional mechanism that contributes to cell cycle arrest. Ubiquitination and degradation of other APC components are known to occur during the checkpoint. In particular, Cdc20 ubiquitination is important to turn over molecules within the MCC and allow new Cdc20 molecules to bind and activate the APC when cells resume cycling (Garnett *et al.* 2009; Foster and Morgan 2012; Uzunova *et al.* 2012). Given the high degree of conservation in the mechanism of SAC-signaling among eukaryotes, it is likely that E2 down-regulation may contribute to the SAC in other systems.

In addition to being down-regulated, the charged form of Ubc1 was also reduced when the APC was inactivated, either by SAC activation or Cdc27 degradation (Figures 2 and 3). The exact consequence of a reduction in charging is not clear. Ubc1 has been shown to assemble ubiquitin chains onto itself in the absence of an E3 (Hodgins *et al.* 1996) and we found that Ubc1 catalytic activity is required for its degradation (Figure 2, G and H). Therefore, one possibility is that when the APC is not able to target substrates, Ubc1 rapidly autoubiquitinates itself, leading to a decrease in the charged form and accelerated degradation. Similar regulation has been observed for other E2 enzymes that undergo increased autoubiquitination when their partner E3s are unable to target substrates. In yeast, Ubc7 undergoes increased autoubiquitination and degradation when its partner E3, Cue1 is deleted (Ravid and Hochstrasser 2007). In human cells, UbcH10 and Ube2S undergo accelerated degradation after all other APC substrates are degraded at the end of G1 phase. However, in the case of UbcH10 and Ube2S, autoubiquitination is APC-dependent (Rape and Kirschner 2004; Williamson *et al.* 2009), whereas in yeast Ubc1 degradation is accelerated when the APC is inhibited, suggesting differences in regulatory mechanisms across different organisms.

Although we find that the catalytic activity of Ubc1 is required for its degradation, mutation of all lysine residues in Ubc1 does not affect its stability (Figure 4, B and C). This is a surprising result, since previous work found that Ubc1 is ubiquitinated on lysines *in vivo* (Swaney *et al.* 2013) and it raises the question of how Ubc1 is degraded. One possibility is that degradation is instead mediated by a ubiquitin chain that forms on the catalytic cysteine, similar to what has been observed for yeast Ubc7 (Ravid and Hochstrasser 2007). Alternatively, since the addition of an N-terminal tag blocks Ubc1 degradation, ubiquitination of the N-terminus could trigger degradation. N-terminal ubiquitination is widespread (Akimov *et al.* 2018) and has been shown to mediate degradation of several proteins in human cells (Breitschopf *et al.* 1998; Bloom *et al.* 2003; Coulombe *et al.* 2004).

Cells have evolved multiple overlapping regulatory mechanisms to coordinate the properly timed entry into and exit from SAC-triggered cell cycle arrest. While the formation of the MCC is the primary effector of the SAC that directly inhibits the APC, it is becoming clear that regulation of additional APC components is also important. Our work suggests that while accelerated degradation of Ubc1 is not essential for the checkpoint, it strengthens the arrest and therefore represents an additional layer of SAC regulation. Interestingly, stabilization of Ubc1 also slows proliferation in otherwise unperturbed cells (Figure 5), suggesting that Ubc1 turnover may also contribute to cell cycle regulation in other ways. Future work investigating Ubc1 regulation in alternate contexts is likely to provide additional insights into this possibility.

Finally, our findings may have implications for treatment of cancers that misregulate E2 enzymes that function with the APC. Both UbcH10 and Ube2S are overexpressed in human cancers (Pallante *et al.* 2005; Berlingieri *et al.* 2007; Ieta *et al.* 2007; Fujita *et al.* 2009; Chen *et al.* 2010; Shen *et al.* 2013; Ayesha *et al.* 2016; Yoshimura *et al.* 2017; Li *et al.* 2018; Pan *et al.* 2018; Lin *et al.* 2019) and elevated expression of Ube2K is associated with a poor prognosis in pancreatic and gastric cancers (Uhlen *et al.* 2017; Wu *et al.* 2020). Although it remains unclear whether Ube2K can function with the APC in human cells, we find that it is able to complement the growth defect in yeast cells after Ubc1 shut-off, whereas Ube2S is unable to do this (Supplementary Figure S4). These findings support the possibility that Ube2K is capable of functioning with the APC in human cells in some contexts. One possibility is that Ube2S may function as the predominant chain-extending enzyme in normal cells, but that Ube2K can acquire that role when it is overexpressed in cancer cells. Our data suggest that cancer cells that overexpress any of these enzymes may be more resistant to treatment with microtubule poisons, similar to what we observe when we overexpress Ubc1 in yeast. In addition, microtubule poisons may act synergistically with drugs that inhibit these enzymes to block cancer cell proliferation.

## Data availability

Strains and plasmids described here are available upon request. The data underlying this article are available in the article and in its online Supplementary Material.

Supplementary material is available at *G3* online.

## Supplementary Material

jkab346_Supplementary_Figure_S1

jkab346_Supplementary_Figure_S2

jkab346_Supplementary_Figure_S3

jkab346_Supplementary_Figure_S4

jkab346_Supplementary_Table_S1
