# Supplementary material for: Ubc1 turnover contributes to the spindle assembly checkpoint in Saccharomyces cerevisiae
Source: G3 (Bethesda). 2021 Sep 29;11(12):jkab346. doi: 10.1093/g3journal/jkab346 (PMC8664427; doi:10.1093/g3journal/jkab346)
Supplement: jkab346_Supplementary_Figure_S1 [file jkab346_supplementary_figure_s1.pdf]

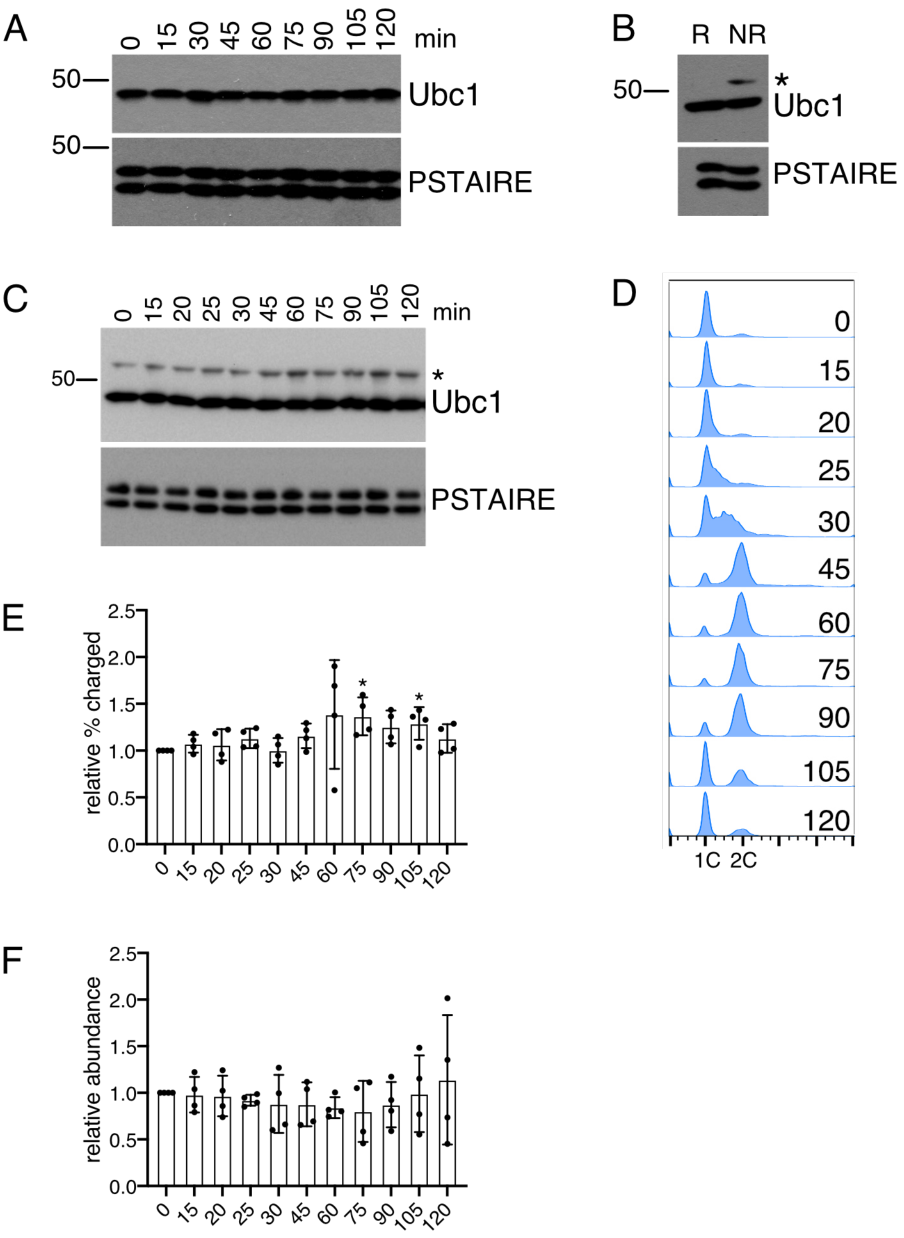

Supplementary Figure S1. Expression of Ubc1 during the cell cycle. (A) Cells expressing Ubc1-TAP were arrested in G1 with α-factor then released into fresh media. α-factor was added back after 45 minutes to trap cells in the second G1 phase. Samples were collected every 15 minutes. Shown are TAP-tag and PSTAIRE (loading control) Western blots. **(B)** Asynchronous Ubc1-TAP cells were lysed and reducing (R) or non-reducing (NR) sample buffer added. Western blots for the TAP-tag and PSTAIRE are shown. The asterisk represents the charged form of Ubc1 that is only evident when non-reducing sample buffer is used. **(C)** Ubc1-TAP cells were arrested and released as in (A) and samples collected at the indicated time points. Non-reducing sample buffer was used for all lysates and Western blotting was performed for the TAP-tag and PSTAIRE. **(D)** Flow cytometry of DNA content from cells in (C), demonstrating synchronous progression through the cell cycle. **(E)** Quantitation of the percentage of charged Ubc1 from (C). Shown is an average of n=4 experiments. **(F)** Quantitation of the relative abundance of Ubc1 (normalized to PSTAIRE) over the course of the cell cycle, as in (C). Shown is an average of n=4 experiments. For (E) & (F) levels are normalized to the 0-minute time point. Error bars represent standard deviations. Paired t-tests were used to determine if any time point was significantly different from 0-minutes, *$p<0.05$.
