# Supplementary material for: Ubc1 turnover contributes to the spindle assembly checkpoint in Saccharomyces cerevisiae
Source: G3 (Bethesda). 2021 Sep 29;11(12):jkab346. doi: 10.1093/g3journal/jkab346 (PMC8664427; doi:10.1093/g3journal/jkab346)
Supplement: jkab346_Supplementary_Figure_S2 [file jkab346_supplementary_figure_s2.pdf]

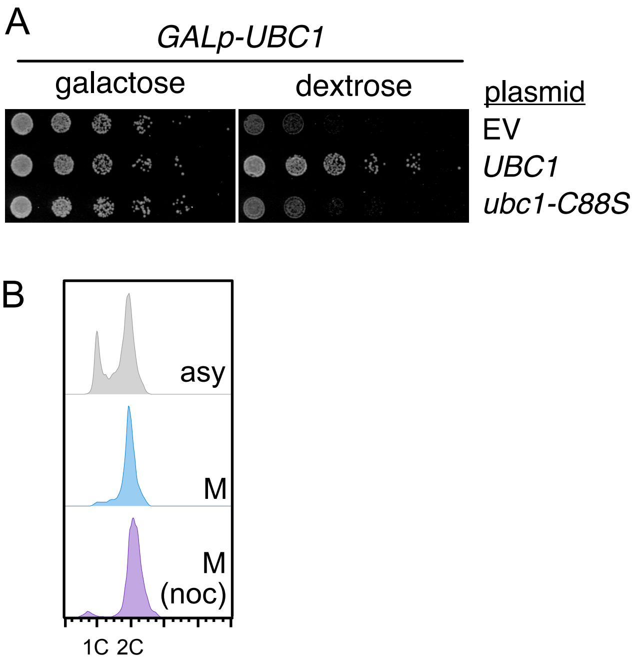

Supplementary Figure S2. Analyses of Ubc1-C88S. (A) Five-fold dilutions of G*ALp-UBC1* cells expressing *UBC1* or *ubc1-C88S* from centromeric plasmids or an empty vector (EV) were plated on C-Ura medium containing galactose or dextrose, as indicated. **(B)** Flow cytometry showing DNA content in cells from Figure 2G-H, to confirm cell cycle position of each culture.
