# Supplementary material for: Ubc1 turnover contributes to the spindle assembly checkpoint in Saccharomyces cerevisiae
Source: G3 (Bethesda). 2021 Sep 29;11(12):jkab346. doi: 10.1093/g3journal/jkab346 (PMC8664427; doi:10.1093/g3journal/jkab346)
Supplement: jkab346_Supplementary_Figure_S3 [file jkab346_supplementary_figure_s3.pdf]

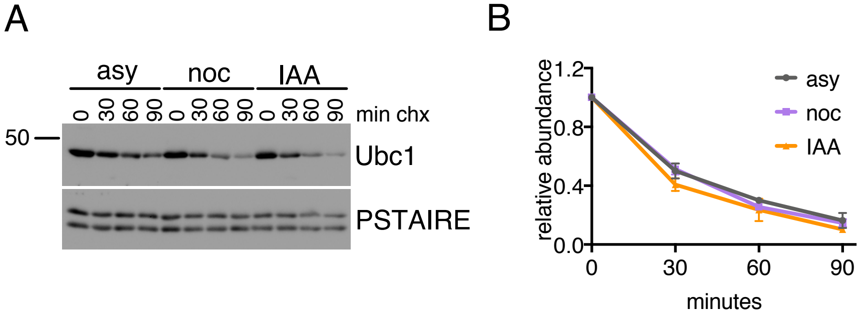

Supplementary Figure S3. Degradation of Ubc1 in CDC27-AID cells. (A) CDC27-AID cells from Figure 3C were treated with cycloheximide following the 3-hour incubation with nocodazole (noc) or IAA and samples taken at the indicated time points. Western blots for Ubc1-TAP and PSTAIRE are shown. **(B)** Quantitation of cycloheximide chase assay from (A). Shown is an average of n=3 experiments. Error bars represent standard deviations.
