# Supplementary material for: Ubc1 turnover contributes to the spindle assembly checkpoint in Saccharomyces cerevisiae
Source: G3 (Bethesda). 2021 Sep 29;11(12):jkab346. doi: 10.1093/g3journal/jkab346 (PMC8664427; doi:10.1093/g3journal/jkab346)
Supplement: jkab346_Supplementary_Figure_S4 [file jkab346_supplementary_figure_s4.pdf]

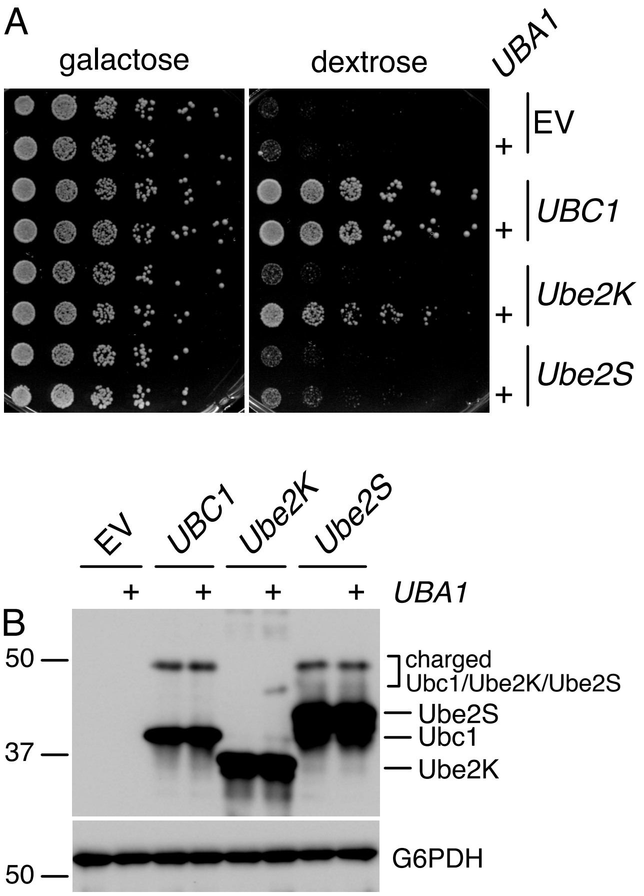

Supplementary Figure S4. Expression of human Ube2K and Uba1 restore growth following Ubc1 shut-off. (A) Five-fold dilutions of *GALp-UBC1* cells expressing the indicated genes from centromeric plasmids were plated on C-Ura-Leu medium containing galactose or dextrose, as indicated. The Ube2K and Ube2S cDNAs were codon optimized to increase expression in yeast. Co-expression of human Uba1 with Ube2K was required to rescue the growth defect. **(B)** Cells from (A) were grown in C-Ura-Leu liquid medium containing galactose and lysed in non-reducing conditions to preserve charged Ubc1/Ube2K/Ube2S. Western blots for the V5 tag on Ubc1/Ube2K/Ube2S and G6PDH (loading control) are shown. Co-expression of Uba1 was required for Ube2K to be charged when expressed in yeast.
