# Supplementary material for: Ubc1 turnover contributes to the spindle assembly checkpoint in Saccharomyces cerevisiae
Source: G3 (Bethesda). 2021 Sep 29;11(12):jkab346. doi: 10.1093/g3journal/jkab346 (PMC8664427; doi:10.1093/g3journal/jkab346)
Supplement: jkab346_Supplementary_Table_S1 [file jkab346_supplementary_table_s1.pdf]

Supplementary Table S1. Strain table.

Strain name	Genotype	Figure
YHA294	*MATa his3Δ1 leu2Δ0 ura3Δ0 met15Δ0 UBC1-3V5-KanMX*	1A
YCL145	*MATa his3Δ1 leu2Δ0 ura3Δ0 met15Δ0 HisMx6-GAL1p-UBC1-3V5-KanMX*	1A
MW839a	*MATα his3Δ1 leu2Δ0 ura3Δ0 lys2Δ0*	1B-C
YCL151-4	*MATα his3Δ1 leu2Δ0 ura3Δ0 lys2Δ0HisMx6-GAL1p-UBC1*	1B-C
YCL181	*MATα his3Δ1 leu2Δ0 ura3Δ0 lys2Δ0 ctf4Δ::KanMx*	1C
YCL162	*MATa his3Δ1 leu2Δ0 ura3Δ0 lys2Δ0 HisMx6-GAL1p-UBC1 ctf4Δ::KanMx*	1C
UBC1-TAP	*MATa his3Δ1 leu2Δ0 met15Δ0 ura3Δ0 UBC1-TAP-HIS3MX*	2, S1, 4B-C
YHA387	*MATa his3Δ1 ura3Δ0 leu2Δ0 met15Δ0 +pRS316-ubc1-C88S-TAP*	2G-H, S2B
YHA390	*MATb his3Δ1 leu2Δ0 ura3Δ0 lys2Δ0 HisMx6-GAL1p-UBC1 +pRS316-UBC1-TAP*	S2A
YHA388	*MATb his3Δ1 leu2Δ0 ura3Δ0 lys2Δ0 HisMx6-GAL1p-UBC1 +pRS316-ubc1-C88S-TAP*	S2A
YMC275	*MATα his3Δ1 leu2Δ0 ura3Δ0 lys2Δ0 ChrVIΔ181901-182001::TIR1-3HA-KanMX*	3A
YHA344	*MATb his3Δ1 leu2Δ0 ura3Δ0 lys2Δ0 CHRVIΔ 181901-182001::TIR1-3HA-KanMX CDC27-AID-6FLAG-HYG*	3A
YHA345	*MATα his3Δ1 leu2Δ0 ura3Δ0 lys2Δ0 met15Δ0 CHRVIΔ 181901-182001::TIR1-3HA-KanMX DBF4-TAP-HIS3MX*	3B
YHA347	*MATa his3Δ1 leu2Δ0 ura3Δ0 lys2Δ0 CHRVIΔ 181901-182001::TIR1-3HA-KanMX CDC27-AID-6FLAG-HYG DBF4-TAP-HIS3MX*	3B
YHA351	*MATa his3Δ1 leu2Δ0 ura3Δ0 lys2Δ0 CHRVIΔ 181901-182001::TIR1-3HA-KanMX CDC27-AID-6FLAG-HYG UBC1-TAP-HIS3MX*	3C-F, S3
YHA326	*MATα his3Δ1 leu2Δ0 ura3Δ0 lys2Δ0 HisMx6-GAL1p-UBC1 +pRS316*	4A, S2A
YHA327	*MATα his3Δ1 leu2Δ0 ura3Δ0 lys2Δ0 HisMx6-GAL1p-UBC1 +pRS316-UBC1-V5*	4A
YHA362	*MATα his3Δ1 leu2Δ0 ura3Δ0 lys2Δ0 HisMx6-pGAL1-UBC1 +pRS316-UBC1-KR-V5*	4A
YHA393	*MATα his3Δ1 leu2Δ0 ura3Δ0 lys2Δ0 HisMx6-GAL1p-UBC1 +pRS316-Myc-UBC1-TAP*	4A
YHA363	*MATa his3Δ1 leu2Δ0 ura3Δ0 met15Δ0 ubc1-KR-TAP-HIS3MX*	4B-C
YHA379	*MATa his3Δ ura3Δ leu2Δ met15Δ HIS3MX-UBC1p-UBC1-TAP-URA3*	4D-E, 5
YHA377	*MATa his3Δ ura3Δ leu2Δ met15Δ HIS3MX-UBC1p-13-MYC-UBC1-TAP-URA3*	4D-E, 5
YHA343	*MATα his3Δ1 leu2Δ0 ura3Δ0 lys2Δ0 HisMx6-GAL1p-UBC1 +pRS316 +pRS415-GPD*	S4
YHA344	*MATα his3Δ1 leu2Δ0 ura3Δ0 lys2Δ0 HisMx6-GAL1p-UBC1 +pRS316-UBC1p-UBC1-V5 + pRS415-GPD*	S4
YHA345	*MATα his3Δ1 leu2Δ0 ura3Δ0 lys2Δ0 HisMx6-GAL1p-UBC1 +pRS316-UBC1p-yUBE2K-V5 + pRS415-GPD*	S4
YHA347	*MATα his3Δ1 leu2Δ0 ura3Δ0 lys2Δ0 HisMx6-GAL1p-UBC1 +pRS316 + pRS415-GPD-UBA1-FLAG*	S4
YHA348	*MATα his3Δ1 leu2Δ0 ura3Δ0 lys2Δ0 HisMx6-GAL1p-UBC1 +pRS316-UBC1p-UBC1-V5 + pRS415-GPD-UBA1-FLAG*	S4
YHA349	*MATα his3Δ1 leu2Δ0 ura3Δ0 lys2Δ0 HisMx6-GAL1p-UBC1 +pRS316-UBC1p-yUBE2K-V5 + pRS415-GPD-UBA1-FLAG*	S4
YHA395	*MATα his3Δ1 leu2Δ0 ura3Δ0 lys2Δ0 HisMx6-GAL1p-UBC1 +pRS316-UBE2S-V5 + pRS415-GPD*	S4
YHA394	*MATα his3Δ1 leu2Δ0 ura3Δ0 lys2Δ0 HisMx6-GAL1p-UBC1 +pRS316-UBE2S-V5 + pRS415-GPD-UBA1-FLAG*	S4
